# Esterified Oxylipins: Do They Matter?

**DOI:** 10.3390/metabo12111007

**Published:** 2022-10-22

**Authors:** Carmen E. Annevelink, Rachel E. Walker, Gregory C. Shearer

**Affiliations:** Department of Nutritional Sciences, The Pennsylvania State University, University Park, PA 16802, USA

**Keywords:** oxylipin, lipoprotein, metabolism, esterification

## Abstract

Oxylipins are oxygenated metabolites of fatty acids that share several similar biochemical characteristics and functions to fatty acids including transport and trafficking. Oxylipins are most commonly measured in the non-esterified form which can be found in plasma, free or bound to albumin. The non-esterified form, however, reflects only one of the possible pools of oxylipins and is by far the least abundant circulating form of oxylipins. Further, this fraction cannot reliably be extrapolated to the other, more abundant, esterified pool. In cells too, esterified oxylipins are the most abundant form, but are seldom measured and their potential roles in signaling are not well established. In this review, we examine the current literature on experimental oxylipin measurements to describe the lack in reporting the esterified oxylipin pool. We outline the metabolic and experimental importance of esterified oxylipins using well established roles of fatty acid trafficking in non-esterified fatty acids and in esterified form as components of circulating lipoproteins. Finally, we use mathematical modeling to simulate how exchange between cellular esterified and unesterified pools would affect intracellular signaling.. The explicit inclusion of esterified oxylipins along with the non-esterified pool has the potential to convey a more complete assessment of the metabolic consequences of oxylipin trafficking.

## 1. Introduction

Oxylipins are oxygenated metabolites derived from, and functionally similar to, polyunsaturated fatty acids (PUFAs) with bioactivities that impact human health and disease. Oxylipin metabolism is physiologically and biochemically similar to PUFA metabolism, but the intricacies of oxylipins signaling expound on PUFA metabolism and can further specify the metabolic processes responsible for health and disease.

This review informs a reader familiar with the bioactivities and metabolism of oxylipins about the complexities of fatty acid compartmentation in esterified and non-esterified forms within plasma, cells, and larger biological systems. It discusses how lipid compartmentation functions in oxylipin trafficking. The intent is that by informing the reader of key details, they can make better analytical decisions and interpretation of results regarding esterified oxylipins. To demonstrate the potential use and meaning of esterified oxylipins, we will: (1) survey the 2020 publication record to evaluate how oxylipins are currently measured, and show the gap in understanding the esterified pool, (2) review fatty acid trafficking in plasma, comparing lipoprotein transport to transport of non-esterified fatty acids (NEFA) and give evidence that plasma oxylipins are trafficked in the same way as plasma fatty acids; finally, (3) review intracellular trafficking of fatty acids, emphasizing the exchange of fatty acids with extracellular lipoprotein and NEFA pools, and use mathematical modeling based on PUFA utilization to demonstrate the potential roles esterified oxylipins could play in cell signaling.

## 2. Current Practice in Oxylipin Measurements

**Figure 1:**
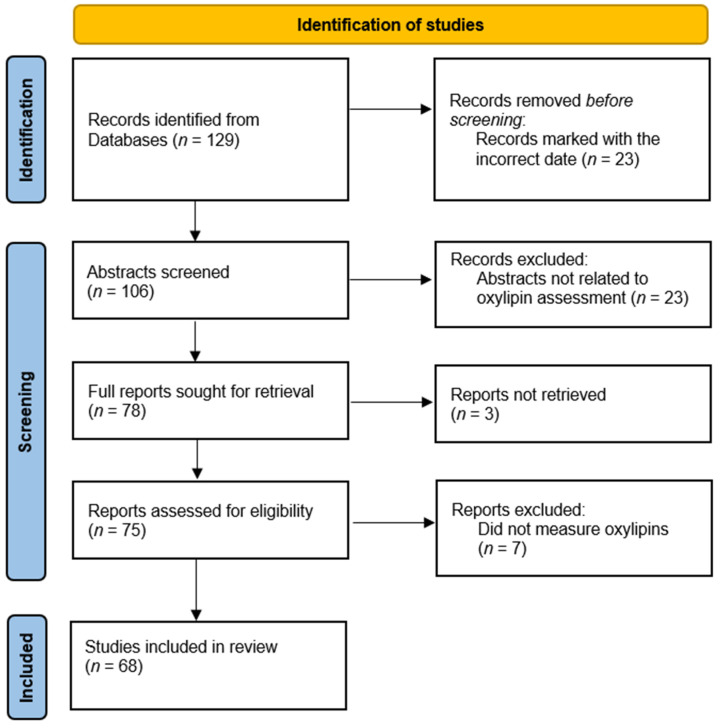
PRISMA Table for Study Identification. PRISMA table shows the process for identifying records to include in our survey of publications, reproduced from the 2020 PRISMA guidelines [25]. Records were pulled from PubMed using “plasma”, “oxylipin”, “oxylipid”, and oxylipin class names (DiHETE, DiHETrE, DiHOME, EpDPE, EpETE, EpETrE, EpOME, HDoHE, HEPE, HETE, HODE, HpETE, KETE, KODE, resolvin, maresin, protectin, EET, EEQ, EEP, DHET, OXO-ODE, OXO-ETE), carried out in humans, limited to the year 2020, and published in English.

*Survey of oxylipin publications*: In a survey of 2020 publications listed in PubMed which measured oxylipins as an outcome (Figure 1), we found that 17.6% of publications include a hydrolysis step to assess esterified oxylipin concentration. Using oxylipin classes as search terms, we found 129 publications, of which 68 reports were assessed as having oxylipin measurements. Of these 68 reports (Table 1), 12 included methods to assess esterified oxylipins [1,2,3,4,5,6,7,8,9,10,11,12]. Of those using a hydrolysis step, most investigators analyzed only total oxylipins, consisting of both esterified and non-esterified, and did not assess non-esterified oxylipins (NEOxs) in parallel to quantify esterified oxylipins. Of the 68 publications, 51 did not have specific terminology regarding the oxylipin pool being measured; typically, the publications identified the oxylipin measured, but not the pool it was measured in. These reports assessed oxylipin concentration in plasma, tissue, cells, and urine from a variety of sample populations including animal and human studies. Ten reports utilized ELISA kits to quantify the levels of oxylipins for their study; of these, 9 were analyzing only specialized pro-resolving mediators (SPMs) [13,14,15,16,17,18,19,20,21,22]. Our survey of the literature returned 7 methodology papers that discussed the protocol for oxylipin extraction as well as the stability and efficiency of extraction procedures in different biological tissues [6,7,8,9,10,23,24]. Of these method reports, 5 included hydrolysis steps to analyze esterified oxylipins and commented on the importance of this biological pool of oxylipins.

*Methods for measuring esterified oxylipins*: Measurement of oxylipins in their esterified form is usually accomplished through the inclusion of a base hydrolysis step followed by incubation of the sample at 60 °C between 30 and 90 min. In order to measure both the total (esterified and non-esterified) and non-esterified pools, the sample must be split and measured with and without hydrolysis. This was not the case in the majority of the surveyed publications, only one publication specified that both the total and the non-esterified pools were independently measured in order to calculate the esterified pool [4]. Notably, an important approach to oxylipin measurement was not apparent in the 2020 literature but represents a powerful methodological tool for oxylipin measurement. Morgan et al. suggested that further separation of oxylipins and analysis of both esterified and non-esterified classes can be completed by combining the traditional, and highly sensitive, LC/MS/MS methods with coupled drift tube ion mobility and high-resolution mass spectrometry (LC/DTIM-MS) [26]. This method utilizes a standard non-esterified oxylipin extraction protocol, without hydrolyzation, and incorporates fragmentation of the sample and analysis of unique mobility behaviors, including collision-cross-section values in nitrogen, to quantify both esterified and non-esterified oxylipins within the sample.

*Implications for measurement*: In summary, unlike methods used to infer intracellular or intra-tissue signaling processes, the compartmentation of oxylipins in plasma suggests that each pool is (1) targeted to unique tissues in the same manner as the lipoprotein and lipid class in which it is located; and (2) is subject to temporal fluctuations based on nutritional status and metabolic condition. As noted, likely reasons esterified oxylipins are rarely measured are experimental efficiency and familiarity; consider that adding this measurement could double the experimenter’s analytical burden. Most labs are familiar with measurement of the non-esterified fraction, and this pool can be measured directly. While a few protocols are sufficient for directly measuring the esterified pool [27], most labs are only set up to measure them indirectly, by subtraction of non-esterified oxylipins from the total fraction. The utility of measuring this pool depends on how independent it is from the commonly measured NEOx pool. Unfortunately, we can provide little practical guidance from the literature.

*What do we lose by not measuring esterified oxylipins*? Having shown that esterified oxylipins are not commonly measured, we next ask: *does it matter?* Since measuring esterified oxylipins most often entails extra analytical steps, the best case for their measurement lies in identifying cases or conditions where they could have a different or unique functional impact. We suggest both intercellular signaling through lipoprotein transport, and intracellular signaling depend on the esterified pool. Therefore, measuring this pool is critical for understanding the physiologic role of oxylipins in the body.

## 3. Esterified Oxylipins in Plasma & Implications

*Fatty acid transport by lipoproteins*: In order to be transported in the aqueous plasma, lipids circulate packaged in lipoprotein particles having a hydrophilic surface of phospholipids and cholesterol, and a neutral lipid core of triglycerides and cholesterol esters. These particles have characteristic apolipoproteins, whose function is to stabilize and direct the particle’s interaction with specific tissues and lipases; hence the term lipoprotein. Phospholipid, triglyceride, cholesterol, and cholesterol esters are the most prominent lipid classes in plasma lipoproteins, however the lipids most relevant to understanding oxylipin transport are fatty acids, which are acylated sub-components of three of these lipid classes: phospholipid, triglyceride, and cholesterol esters.

Lipoprotein biology is complex and under constant fine-tuning by field experts. A full description is beyond this review’s scope, but some valuable overviews are available [28,29,30,31], themselves pointing to, or referenced by, other excellent and more detailed descriptions. Plasma lipid transport is most commonly conceptualized around the direction of cholesterol movement. Because of this emphasis, it is easy to overlook other primary lipids transported by lipoproteins. In general, the direction of fatty acid movement is similar to cholesterol, and by implication so is the direction of oxylipin transport. The fatty acid components of lipoprotein lipids are uniquely targeted to tissues based on: (1) the lipid they are acylated into, and (2) the lipoprotein they are located in. Fatty acids supplied from the diet, newly synthesized from de novo lipogenesis, or through lipolysis of intracellular stores [32] are transported in four forms: as triglycerides, predominantly in chylomicrons (CM) and Very Low-Density Lipoproteins (VLDL), minorly in Low-Density Lipoproteins (LDL) and High-Density Lipoproteins (HDL); as phospholipids predominantly in HDL, less so in LDL, and minorly in CM and VLDL; as cholesterol esters in LDL and HDL; finally in non-esterified form (see below).

*Forward cholesterol transport* commonly describes the transport of lipoproteins originating in the liver (VLDL/LDL) and small intestine (CM/CM-remnant). ApoB containing lipoproteins from the liver (apoB-100) and intestine (apoB-48) are loaded with lipids, primarily triglycerides, and distributed into the circulation where they are subject to the action of lipoprotein lipase (LpL). LpL hydrolyzes lipoprotein triglycerides and esterified fatty acids in triglycerides are released at the vascular endothelium and diffuse to underlying tissues. LpL hydrolysis leaves behind a remnant core of phospholipid, cholesterol, and cholesterol-esters which are ultimately transformed into a cholesterol-rich LDL particle, and are available to tissues expressing the LDL-receptor (LDLr).

*Reverse cholesterol transport* describes lipid transport originating in tissues peripheral to the liver. ApoA-I comprises the central structural apolipoprotein which removes cellular lipids predominantly via ABCA1, ABCG1, or SR-B1-dependent exchange [33]. Following cholesterol removal, HDL mature by lecithin-cholesterol acyl transferase (LCAT)-dependent cholesterol esterification and lipid exchange with apoB lipoproteins by the action of cholesterol-ester transfer protein (CETP) or phospholipid transfer protein (PLTP) ultimately returning much of the cholesterol to the liver.

*Enzymes and apoproteins target each lipoprotein lipid*: One of the unique features of plasma lipoproteins is that each lipoprotein and each lipid class is uniquely trafficked. As described above, the cholesterol in LDL has a destination distinct from the cholesterol in HDL, and this distinction is reflected in the common reference of HDL-cholesterol as the “good cholesterol”, and LDL-cholesterol as the “bad cholesterol”, arising from the pathological role of LDL to deliver cholesterol to atherosclerotic plaques as a part of its forward transport role, and of HDL to remove cholesterol from atherosclerotic plaques as a part of its reverse transport role. Molecularly, the biggest factor determining the destination of LDL-cholesterol is expression of the LDLr. Tissues expressing the LDLr are capable of removing circulating LDL and their cholesterol-ester content [30,34]. In like manner, the destination of triglyceride in VLDL is determined at the tissue level by expression of LpL, but also by the apolipoprotein complement of VLDL, including apoE, which facilitates binding of VLDL to heparan sulfate proteoglycans on the surface of endothelial cells.

*Trafficking of non-esterified fatty acids*: NEFA are available to cells interacting with dissolved small molecules in plasma, similar to glucose or amino acids, with the exception that their solubility in plasma is stabilized by binding to albumin [35]. Nutritional status is the primary regulator of NEFA availability. Following a glucose containing meal, an increase in plasma insulin concentration results from the glucose-mediated insulin appearance. In turn, adipocytes respond to insulin by suppression of intracellular lipolysis, the rate of NEFA appearance into plasma drops precipitously, and the concentration of circulating NEFA declines rapidly, averaging over 75% suppression by 60 min in optimally healthy individuals [36]. This insulin effect to suppress circulating NEFA allows for efficient glucose utilization by limiting the contribution of fatty acid β-oxidation to mitochondrial acetyl-coA, and facilitating the provision of glycolysis to contribute acetyl-coA via pyruvate dehydrogenase, a phenomenon commonly termed the Randle Cycle [37].

It is not clear whether NEOx are similarly made available by intracellular lipolysis in adipocytes. While the majority of studies measure circulating NEOx, most samples are obtained from participants in the fasting state where the most likely drivers of NEFA are counter-regulatory hormones, not insulin. We are aware of only one that measures NEOx in response to a meal or glucose challenge [38] in a single individual, and the data were consistent with insulin-driven suppression following a meal, and a high fasting steady-state driven by counter-regulatory hormones. Follow-up studies would greatly assist in understanding the extent which circulating NEOx share a common origin with NEFA.

*Lipoprotein trafficking of oxylipins*: Relatively little is known about the specific trafficking and transport features of oxylipins. They are esterified into the same lipids as fatty acids, since they retain their α-carboxylic acid. For this reason, the transport and trafficking of FAs, and of PUFAs in particular, is a reasonable starting point for understanding the basis of oxylipin trafficking. In circulation, oxylipins can be found in their non-esterified form and bound to proteins, but the majority of oxylipins (~90%) are found esterified to cellular lipids and in lipoproteins [39,40], and each lipoprotein transports a specific complement of oxylipins [41,42]. There is evidence in the literature for using lipoprotein transport of fatty acids as a framework for understanding oxylipin transport by lipoproteins. Oxylipins in VLDL are transported to the same tissues that consume VLDL triglycerides—tissues that express LpL or the VLDL receptor including skeletal muscle, myocardium, and adipose. The liver is able to actively package oxylipins into VLDL [43]. In response to an inflammatory challenge, the liver actively increases the rate of circulating non-esterified linoleic acid removal, elongates it to arachidonate (AA), makes epoxyeicosatrienoic (EpETrEs) and hydroxyeicosatrienoic acids (HETEs), and packages them into VLDL [43]. The lipolytic action of LpL acts on oxylipins esterified into VLDL lipids in the same way as it acts on esterified fatty acids [40]. Only indirect evidence for specific trafficking of LDL or HDL oxylipins exists. Murphy et al. have shown that activation of Ffar4, a regulator of intracellular phospholipase A_2_ (PLA_2_) activity, in macrophages increases cell 18-HEPE content, and mice lacking Ffar4 lack 18-HEPE in their HDL [44]. Changing nutritional status [45,46] or intake of PUFA impacts the abundance of oxylipins in lipoproteins [42,47].

*Intercellular signaling* of oxylipins has been well characterized by Sala et al. where the eicosanoid biosynthetic pathway is shown to be an important contributor to production of prostaglandins, leukotrienes, and lipoxins in the body [48]. Platelet-derived PGH_2_ requires endothelial cells for uptake and conversion of PGH_2_ into PGI_2_. In isolated organ preparations, neutrophils adhere to coronary endothelial cells and are associated with LTC_4_ production and an increase in coronary perfusion pressure—an effect that was inhibited when pre-treating the neutrophils with an inhibitor of 5-LOX activating-protein [49,50]. In a similar function, lipoproteins in the plasma could act as one side of the described intercellular signaling and are critical for providing and transporting precursor oxylipins not present in other cells or tissues and allowing for downstream modification of oxylipins.

As noted, most NEOx in circulation are not “free”, but are bound to plasma albumin [51], hence the true concentration of unbound oxylipins available for binding to receptors is actually much lower than measured. This should especially be considered in pathologies where plasma albumin is low (e.g., nephrotic syndrome, hepatic disease) or where albumin binding is impaired (e.g., uremic syndrome) [52].

*Implications for circulating oxylipins*: A large portion of studies report on circulating oxylipins, reflecting their potential usefulness both as a biomarker or as a means to understand physiological and pathological adaptations (see earlier section). While much insight has been gleaned, a concerning element of reporting occurs when the status of circulating oxylipins measured is not communicated. In some cases, circulating NEOx are represented as the entirety of circulating oxylipins.

Leveraging the complex compartmentation and destinations of circulating esterified oxylipins, how the various pools are subject to regulation based on nutritional status, metabolic status, and pathologically induced changes could add great detail to the understanding of oxylipin biology and their specific regulatory roles. While many factors underly the decision to measure non-esterified or esterified oxylipins, the decision is a critical element of study design, and the activity of interest should be a factor in the decision. Compartmentation of oxylipins among pools in circulation immediately introduces a challenge in experimental measurements. In most cases, resources limit investigators from querying all possible pools. Even where resources are available, measuring multiple pools can make post hoc analysis burdensome. A recommended strategy is to consider the target tissue’s access to fatty acids and the nutritional states most likely to affect the outcome of interest. Does the tissue obtain fatty acids by NEFA or by VLDL? Under what metabolic states is that access limited or facilitated? For example, the myocardium constitutively expresses LpL and obtains most fatty acids from VLDL. In contrast, skeletal muscle primarily obtains fatty acids from the albumin-bound NEFA pool in the resting state, but expresses LpL when demand for β-oxidation increases. Hence, a good starting point for understanding forward delivery of oxylipins to myocardium would be VLDL, while both NEOx and VLDL could deliver oxylipins to skeletal muscle in a metabolic condition-dependent manner. Understanding how esterification imparts biological function can improve sampling decisions and data interpretation.

A second consideration is simply the nature of dyslipidemias: for example, the altered VLDL and HDL of the metabolic syndrome naturally raises the question of how oxylipin delivery and exchange is altered in the condition. Understanding how dys-oxylipinemias contribute to the pathologies of dyslipidemias is a promising way to provide molecular associations with inflammatory disease, especially when cholesterol transport alone is a poor explanation.

## 4. Esterified Oxylipins in Cells & Implications

**Figure 2:**
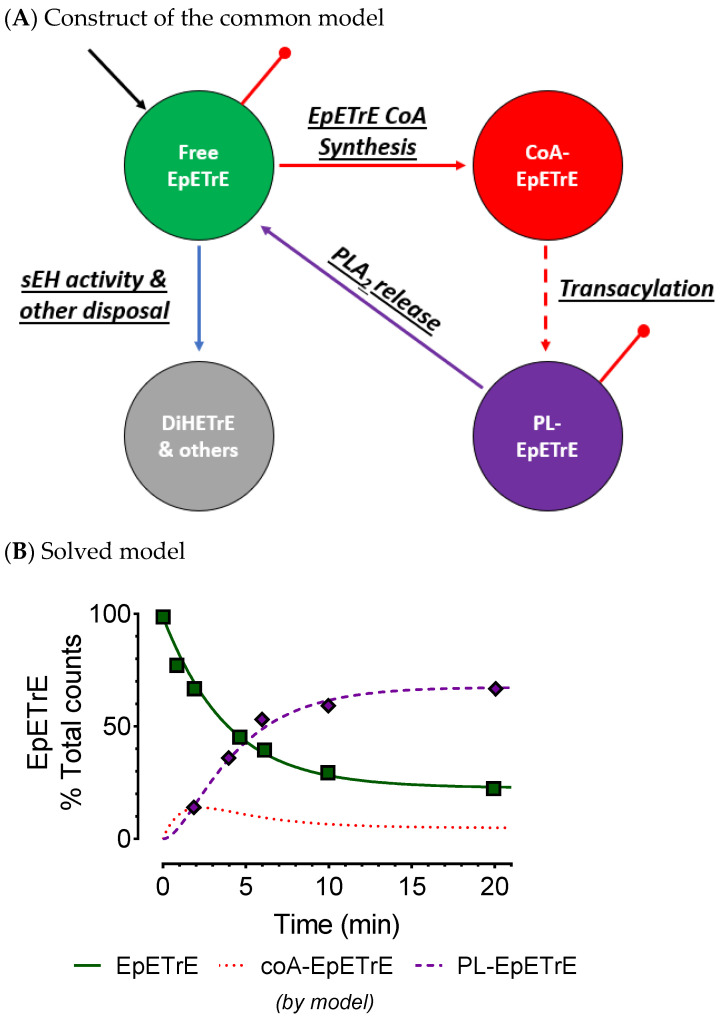
Extant data confirm active role for esterification. An expression of the common model is shown demonstrating the role of intracellular esterification of EpETrE (**A**); components modeled here are shown color coded for (**A**,**B**). Input into the green compartment (black arrow) represents novel EpETrE synthesis. Esterification into PLs is a two-step process: thioloization into coA-EpETrE (red compartment), and acyltransferase activity with lyosophospholipids to create a phospholipid-EpETrE (purple compartment). Disposal of EpETrE occurs as sEH activity from the cytoplasm (green compartment) and is represented by the blue arrow. Red dots represent directly observed data. (**B**): To demonstrate the relative role of esterification versus ‘inactivation’ of EpETrE by sEH hydrolysis and other means, a model was constructed from extant radioactive isotope incorporation data from Karara et al. Model fit of the data using Bayesian Information Criteria is good and it conforms to the conclusions of Karara et al. The model indicates epoxide sequestration in membrane phospholipids, not disposal of free (e.g., by sEH), are the major route for signal termination. Rate constants and associated half-lives are reported in Table 2. CoA, coenzyme A; DiHETrE, dihydroxyeicosatrienoic acid; EpETrE, epoxyeicosatrienoic acid; PL, phospholipid; PLA_2_, phospholipase A-2; sEH, soluble epoxide hydrolase.

*Intracellular esterified oxylipins*: Few studies in tissues or cells explore esterified oxylipins. Early studies report their presence in tissues acylated into the glycerolipids of renal [53], hepatic [54], cardiac [55], lung [56], and more [57], and have been detected in the phospholipid and triglyceride pools [58] although they are present in other glycerolipid pools as well. 12-HETE is present in eosinophils almost entirely in phospholipids and triglycerides [58]. Few longitudinal measures of cellular oxylipins are available—a number of reports include time-dependent data from phospholipid oxylipins, however we identified three with extant, time-dependent data describing the distribution of epoxides and alcohols among cell esterified and non-esterified compartments [24,30,31]. While each use the common, Lands cycle, model of PLA_2_-mediated release and recycling (Figure 2) [59] as a guide for oxylipin signaling, none employ formal compartmental modeling. Without formal modeling it is difficult to assess whether recycling of oxylipins occurs, the prevalence of recycling relative to novel synthesis of oxylipins from AA, or to predict how the system might respond to stimuli or perturbances. Experiments using radioactive isotope incorporation into rat liver tissue explants by Karara et al. [53] had data of sufficient quality and nature for kinetic modeling. We digitally extracted the data from the original manuscript using a data digitizer (automeris.io/WebPlotDigitizer/) and used the extant data to construct a compartmental model applying the EpETrE data to the construct in Figure 2. This allowed us to evaluate the common model’s validity for EpETrEs and demonstrate how the esterified and non-esterified EpETrE pools are likely to interact. The excellent fit of data to the model confirms the validity of the common model and describes a system with rapid uptake and incorporation of EpETrE from the non-esterified pool into phospholipids, followed by a slower, PLA_2_-mediated release to the non-esterified pool (Table 2). The slowest step is the removal of EpETrE from the non-esterified pool by either soluble epoxide hydrolase (sEH) or by other mechanisms. This step was not well resolved from zero, but we estimate the rate for esterification into phospholipids is approximately 30-fold greater than that of the final inactivation rate, and the result is a system that undergoes substantial recycling of EpETrE back and forth between phospholipids and the non-esterified pool, about 12 times in total. This means PLA_2_ directly releases oxylipins; moreover, at any time the majority of oxylipins in the non-esterified pool appear there by direct, PLA_2_-mediated hydrolysis of oxylipins from membrane phospholipids (aka oxylipin recycling), not from PUFA-driven oxylipin synthesis.

**Figure 3:**
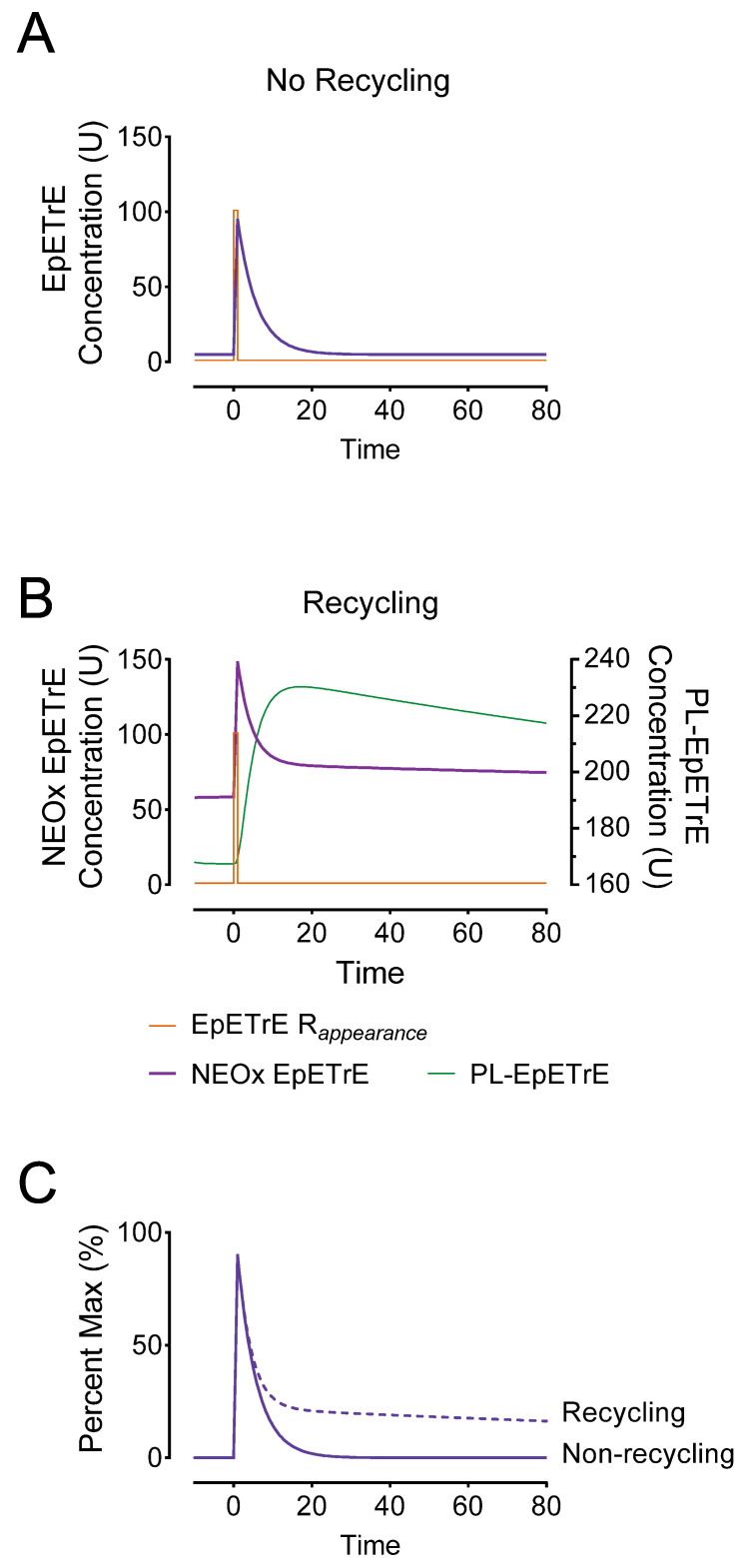
Comparison of non-recycling systems to recycling systems. To graphically illustrate the potential consequences of sequestering EpETrEs into membrane phospholipids, we developed a simulation based on the model in Figure 2. We simulated both basal EpETrE synthesis at 1 U/min and an adaptive response to stimulate EpETrE production, as a 100-fold increase in EpETrE production to 100 U/minute beginning at time = 0 and lasting for 1 min (EpETrE R_appearance_). Model (**A**) represents the system with no recycling in which all of the fractional clearance (FCR = 0.21) is from the cytosolic NEOx to permanent disposal, as would be the case if EpETrE disposal occurs entirely in the cytosol by sEH-mediated hydrolysis to vicinal diols. Model (**B**) represents the system with recycling, in which the fractional clearance from the cytosol is divided as 0.19 pools/min to coA-EpETrE and 0.02 pool/min to sEH and other disposal. The total fraction clearance from the cytosol is identical, however most occurs by re-acylation into membrane phospholipids and only a fraction by sEH. Panel (**C**) overlays NEOx from both systems, the non-recycling (solid) and recycling (dashed) after subtracting out background differences in steady state from each. This facilitates visualization of the effect recycling has on signal extension. The simulated models are theoretical in nature and concentrations are in arbitrary units (U).

*The implications of recycling on oxylipin signaling*: To demonstrate how recycling would impact systems undergoing transient adaptive stimuli, we used the model as a basis for simulating an activation event (Figure 3). This was simulated at 0 min using a rapid 1-min appearance (or input) of EpETrE at a rate of 100 units/minute over a basal EpETrE appearance of 1 U/min. We compared the response to this stimulus in a system lacking recycling to a system with recycling. In both systems the net fractional catabolic rate (FCR) was 0.21 pools/min; in the non-recycling system, the net FCR from NEOx was 0.21 pools/min, but in the latter the FCR was similar to the estimates in Table 1: we set the FTC from NEOx to EpETrE-coA at 0.19 pools/min; since the FTC to disposal in the Karara model was unstable (i.e., sEH activity), we set it to the upper 95% bound, 0.02 pools/min. In the system lacking recycling (Figure 3A), the model predicts a low steady-state EpETrE concentration (5.8 U) followed by a rapid increase in cytosolic EpETrE, peaking at 95 U. In contrast, the system having recycling (Figure 3B) was characterized by a much higher steady-state concentration at 58 U, and a higher peak EpETrE concentration of 149 U, and most prominently, a highly extended signal duration (Figure 3C).

**Figure 4:**
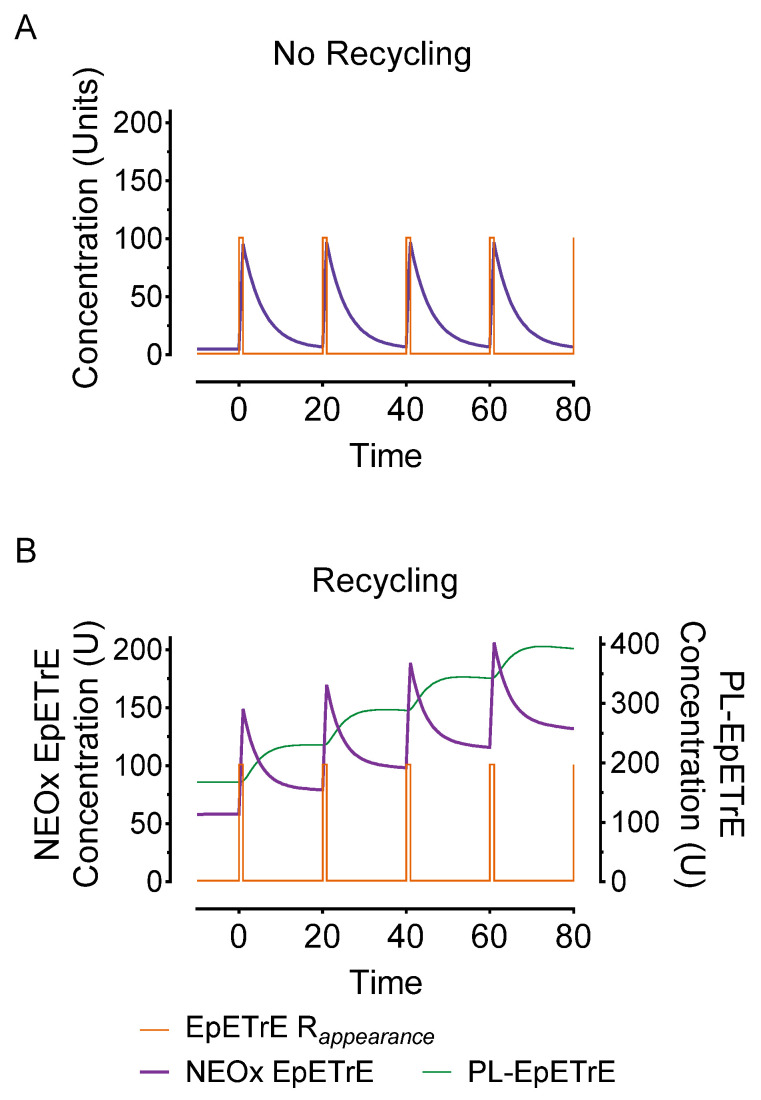
Effect of repeated stimuli on non-recycling (model **A**) and recycling systems (model **B**). To illustrate the impact of successive intervals of EpETrE appearance, the simulations in Figure 3 were modified to simulate repeated pulsed appearance of EpETrE (100 U over 1 min) every 20 min (EpETrE R_appearance_). This feature demonstrates how a recycling system (model **B**) is better organized for signal amplification in response to successive stimuli. As in Figure 3, concentrations are in arbitrary units (U).

We summarize features of the simulation that differentiate the recycling system and could be useful in guiding studies measuring esterified oxylipins in cells. Important characteristics include:

1. *Steady-state*: Compared to a non-recycling system, the basal steady-state concentrations in recycling system producing 1 U/min EpETrE were much greater: 5.8 U vs. 58 U. This drastic difference is largely a function of the permanent disposal, and indicates that in cells undergoing basal oxylipin synthesis, steady state levels are a function of net disposal (e.g., sEH activity), not recycling.
2. *Rapid (but incomplete) signal termination:* Recycling allows for rapid termination of most EpETrE activity in the cytosol. In the non-recycling systems where all disappearance is by cytosolic degradation, the half-life is 3.3 min with the signal nearly 50% terminated by 14 min; however, in the recycling system, the initial rapid half-life is identical and the signal is 50% terminated at nearly the same time.
3. *Signal extension:* Despite rapid initial termination, signal duration in the recycling system is paradoxically extended. This is because most EpETrE disappearance is re-acylation, not sEH-mediated hydrolysis. The half-life for disposal of EpETrE by sEH hydrolysis is 34 min. After 10 min recycling becomes the dominant process, extending the presence of EpETrE in the cytosol so that it takes approximately 235 min until 10% of peak concentration remains, well beyond the modeled timeline.
4. *Stronger signal summation:* Finally, recycling would facilitate signal summation. Successive stimuli of EpETrE production would summate more rapidly than systems lacking recycling. Figure 4 depicts the same stimulus as in Figure 3 (100 units over 1 min) but repeated every 20-min. Repeated stimuli do not accumulate in the non-recycling system due to rapid, irreversible disposal. However, in the recycling system, the successive stimuli accumulate in the phospholipid membrane and facilitate a consecutively greater and greater amount of EpETrE in the cytosol with each successive stimulus.

PLA_2_ plays a central role in facilitating oxylipin recycling in addition to its traditional role of providing substrate for oxylipin production. Notably, in cases where the membrane oxylipin content is low, PLA_2_ activity is likely to have a minimal effect on NEOx since the amount of stored oxylipin in the membrane are low and oxylipins in the cytosol would be more directly related to the rate of oxylipin production by oxylipin producing enzymes. However, under conditions where the concentration of esterified oxylipins is high, PLA_2_ activity would have a large effect on NEOx and activation would result in substantial increases in non-esterified EpETrE, independent of the rate of appearance by CYP*_epox_* activity. This prediction has been demonstrated experimentally: loading of EpETrE into the phospholipids of coronary artery endothelial cells potentiates relaxation of porcine aortic rings upon activation of PLA_2_ [61]. The prediction extends to lipoxygenase metabolites as loading of cells with mid-chain alcohols, where the response of polymorphonuclear leukocytes to agonist-induced activation of PLA_2_ was dependent on whether 5-HETE or 15-HETE was loaded [62]. The compartmental model used here is based on explant culture systems, as are the experimental examples, and many aspects of in vivo systems are not fully in place. Regardless, the aim of the exercise is to demonstrate the system’s functional dynamics sufficiently to reveal the potential biological roles of each pool and the conditions under which each pool should be measured.

## 5. Conclusions

We have provided an estimate of the focus of contemporary studies using oxylipins as predictors, mediators of physiology or pathology. We show a strong current emphasis on NEOx in plasma. Little attention is focused on the most abundant pools of circulating oxylipins in lipoproteins, specifically chylomicrons, VLDL, LDL, and HDL—each of which represent oxylipins targeted to specific tissue and cell classes—and opportunities to improve understanding of lipoprotein oxylipins are suggested. A minority of work is focused on esterified oxylipins and their intracellular bioactivities. We describe a working theory for how recycling, similar to the Lands cycle, could explain the biological function of oxylipins esterified in cell phospholipids. It is our hope that these theories will help improve experimental planning, analysis, and interpretation of oxylipin trafficking.

## Figures and Tables

**Table 1:** Summary of 2020 oxylipin publications.

	Hydrolysis	No Hydrolysis	
Location			ELISA
**Plasma**	10	38	8
**Tissue**	0	9	1
**Cell**	2	5	1
**Urine**	2	0	0
Total *	12	46	10

* Some papers measure multiple locations.

**Table 2:** Kinetic parameters for published epoxide models. Karara et al. studied the incorporation of labeled free epoxide as 1-^14^C 14(15)-EpETrE, into phospholipids of microsomal fractions from homogenized rat livers [60]. The fraction of label in free epoxides and phospholipid-epoxides were measured by liquid scintillation for several time points over 20 min. By 20 min, the fraction of label in phospholipids was stable at about 70%, indicating a steady state. The data were digitally extracted and used to construct a mathematical compartmental model and calculate the kinetic parameters presented here (see Figure 2 for construct and model fit). The calculated recycling number is the average number of times a labeled molecule was recycled through the system [residence time/transient time −1] when entering the system from compartment 1, where residence time is −1 × (the value from the inverse matrix). CoA, coenzyme A; PL, phospholipid; PLA_2_, phospholipase A-2; sEH, soluble epoxide hydrolase.

Enzyme Activity	Phospholipid Data Mean (95%CI)
Acyl-CoA Synthesis (pools/min)	0.19 (0.16, 0.21)
Esterification (pools/min)	0.82 (0.58, 1.06)
PLA_2_ Release(pools/min)	0.065 (0.048, 0.082)
sEH (pools/min)	0.0064 (−0.0046, 0.0173)
Residence Time in Free (min)	157
Residence Time in coA (min)	36
Residence Time in PL (min)	450
Total Time in system (min)	643
Recycling Number	29
